# Proceedings Mississippi Valley Dental Association

**Published:** 1882-04

**Authors:** E. G. Betty


### PROCEEDINGS.

### MISSISSIPPI VALLEY DENTAL ASSOCIATION.

### Discussions of the Thirty-Eighth Annual Meeting of the Mississippi Valley Dental Association at Cincinnati, March 1, 2, and 3, 1882.

(Reported by E. G. Betty, D.D.S.)

The thirty-eighth annual meeting of this association was called to order by President J. W. Jay. The Executive Committee presented the following programme; each subject introduced by a paper:

I. Definite Causes of Dental Caries.

J. S. Cassidy, M.D., D.D.S.

II. Microscopic Conditions of Dental Decay.

J. Taft, M.D., D.D.S.

III. The Dental Pulp, its Pathology and Treatment.

M. H. Chappel, D.D.S.

IV. The Possibilities of Success in the Treatment of Pulpless Teeth and Alveolar Abscess. A. O. Rawls, D.D.S.

V. Filling Teeth with Cohesive Foil.

E. G. Betty, D.D.S.

After the transaction of routine business by the Association, Dr. J. S. Cassidy read his paper on the first subject of the programme.

Dr. Berry remarked that for some years past, there had been a disposition on the part of the members to hold back, and not start the discussion of the subject that had been presented. It will do the society but little good if the members do not at once push to the front and give life and energy to the matter in hand. He noticed this lack of enthusiasm at the present opening of the meeting. Dr. Cassidy's paper is an excellent one, and one containing many points of value, and is worthy of thorough consideration.

Dr. Osmond took exceptions with Dr. Cassidy in the statement that acids are the decay-producing agents. His experience led him to believe that alkalies are in a great many instances responsible for the decay.

In many mouths that are cleaned by the continual use of soap, he had noticed a softening of tooth-structure that resulted in the ultimate destruction of the teeth. The *modus operandi* of the decay as produced by his alkaline theory he did not explain further than that the alkali united with the animal portion of the tooth.

Dr. J. Taft emphatically pronounced Dr. Osmond's theory as radically incorrect. So far as soap is concerned, it is a substance the mutual affinity of whose elements are satisfied with each other. The rapacity of the alkali having been expended in the saponification of the oil, fat or grease composing the soap.

Adjourned till afternoon.

### FIRST DAY—AFTERNOON SESSION.

The discussion of the first subject was resumed.

DR. WRIGHT, upon being asked what was the idea of dentists in Europe upon the causes of caries, replied that some are in favor of one theory, others indorse another. There exists among them the same diversity of opinion that obtains in the United States. In certain localities in Europe, as for instance Tuscany, where the soil is of volcanic nature, the teeth of the people are of good quality. This is due, in all probability, to a larger proportion of phosphates in the soil.

DR. G. W. KEELY.—Dr. Wright, how do the teeth in Europe compare with those in America?

DR. WRIGHT.—It appears that in Southern Germany and Switzerland the teeth are white and soft, and in consequence decay progresses more rapidly.

DR. J. TAFT.—The principles upon which the treatment of decay depend are being modified all the while. There is a growing disposition to combat decay by general systemic treatment, rather than by a sole reliance upon filling, as formerly. The operation of filling is now a great deal more efficient in its details and results than it was some years ago; yet it alone should not be altogether our only treatment.

The general inquiry now is, what can I do to arrest decay of the teeth? this is the gist of a large part of the discussions in societies at the present time. By general treatment the aim is to prevent fermentation, decomposition of unstable bodies and the vitiation of the secretions of the mouth. The operation of decaying agents can be readily understood and expressed in definite terms. All decay is caused by newly formed agents. Acids introduced in the mouth in food, drink or otherwise, do not produce true decay; they merely cause a corrosion that is in no sense allied to true dental caries. The immediate or definite causes of dental caries are right on the spot and act immediately. The saliva contains alkaline materials, but there are other sources of origin. The expired breath contains a greater or lesser amount of ammonia, as the case may be. The fluids of the mouth, by absorbing this ammoniacal gas of course become alkaline in proportion to the amount absorbed. The test applied to the breath itself will demonstrate the presence of ammonia. When, then, this ammonia is oxidized in the mouth, nitric acid in its nascent state is developed, ready to act immediately. The irritation of the mucus membrane at the margin of the gum will cause a vitiated and acidulated secretion in direct contact with the neck of the tooth. The tooth at this point not being supplied with an impervious protecting enamel, at once succumbs to the rapacity of the acid. The decay may be more or less vigorous, either one or other or both of the animal and mineral constituents may be destroyed and washed away. If the irritation at the margin of the gum be due to deposits of foreign bodies, their removal is indicated. It is useless to cauterize the margins of the gum, or the sensitive dentine underlying. Much more is to be gained by treating constitutionally, than mere topical measures. Simply filling the part destroyed only palliates but does not radically alter the condition of things, as a few months time shows softening and a recurrence of the decay about the filling.

Dr. Wright.--It is a fact that the Swiss living in the valleys are edentulous at middle life. Those living at an elevation of 3,000 feet possess good teeth.

Dr. J. Taft.—In the valleys, almost anywhere, the circumstances are adverse to a good development of the body generally, and of course of the teeth. Have seen quite a number of people living in the Blue Grass region of Kentucky, and they all are the possessors of excellent teeth. The animals bred in that region are supplied with a good bony structure. Would like to know if these facts are merely coincident or are the result of superior surroundings.

Dr. Wilkes Smith, upon being called for, stated that as a rule, the teeth of people in the Blue Grass region are better than those in lower countries. However, one can scarcely draw comparisons in that way. The habits of people exert a great modifying influence upon the teeth. The better and more intelligent classes of people pay more attention to cleanliness and hygiene, and as a result present a better quality of teeth. Cistern water

(he possibly meant well water) is mostly used, and probably contains a considerable amount of phosphates. An American, when seen abroad, if he is tall, large and well formed, is usually said to be a Kentuckian. It is very difficult to say what effect climate has upon the teeth.

Dr. H. A. Smith said that Dr. Wright's statement that cereals raised at high elevations is good, undoubtedly, more phosphates being present. Acids confined to one locality can be tested by litmus paper and proved to exist, though they could not be tasted. On the other hand, acids conveyed to the mouth can at once be detected by the sense of taste

Dr. Jas. Leslie cannot see what reason there is for believing that an acid formed in the mouth should necessarily always attack tooth structure. He would like to have a statement made as to how hydro-chloric, nitric and lactic acids are formed in the mouth.

Dr. J. Taft, acids occurring in the mouth are formed just exactly as acids are formed anywhere else. Chemical elements combine in definite proportions, and where one body undergoes decomposition, new combinations immediately succeed. The decomposition of a nitrogenous substance liberates ammonia, and it is the union of this with oxygen that produces nitric acid. Nobody can taste it, but that is not evidence that it is not there. All the elements are present, and when under favorable circumstances their affinities will assert themselves, anything else to the contrary notwithstanding. The affinity of oxygen for any other body will always result in a union. Dr. Leslie forgets that the acid causing decay is in the nascent state, and not as having existed for some time and then introduced into the mouth.

Dr. Kempton.—Dr. Leslie is either remiss in his knowledge upon the point at issue, or is laboring under a mistake. He speaks of an acid going to the tooth. Such is not the case. Food lodges between the teeth or around them, and when decomposed liberates its elements right in contact with the tooth. Just at the moment of formation an acid is in the nascent state.

Dr. Clayton. If theoffice of the acid is to remove devitalized parts, then why is it that devitalized tissue is found only in approximate surfaces and the fissures of the grinding surfaces. A passive or negative cause of decay is by the dissociation of enamel prisms by friction in a lateral direction during mastication. The retention of food between the teeth undoubtedly does cause decay, but whether it is by decomposition and formation of gases, he does not know.

DR. CRAVENS.—The discussion seems to have taken the direction of chemistry, and that is a science about which he said he knew but little. He believes that Dr. Watt is right in his theory of decay. Dr. Watt was at one time accused of being crazy, because he announced that dental caries is only the result of chemical action. According to Dr. Watt, the saliva may be decidely alkaline if a salivary gland is stimulated. Dr. Cravens said that when he took chlorate of potash into his stomach in fifteen or twenty minutes after, when the taste had left the mouth, his saliva would become so alkaline that the taste again became apparent. This illustration was given to demonstrate the fact that the saliva, and, consequently, the fluids of the mouth are not always in the same state chemically, things taken into the system frequently affecting the character of oral fluids in a few moments, time. Acids in their nascent condition are sure to get in their work on the teeth. The active agent of brown decay is hydro-chloric acid; of the white, nitric acid; of the black, sulphuric acid. Black decay, when examined, will be found to extend over a superficial area, with a point of deep penetration. The brown decay penetrates to the pulp without much lateral spreading. The white decay pays no respect to either the animal or mineral constituents of the tooth. He has seen no cases of exposure of the pulp by black decay. That occurs frequently with brown decay and most often under the white. The enamel is only attacked by the white decay.

Adjourned.

### SECOND DAY—MORNING SESSION.

The discussion of the first subject having been concluded, Dr. M. H. Chappel read a lengthy paper on the "Dental Pulp, Its Pathology and Treatment." (*See first article in this number.*) Its discussion then followed.

Dr. Berry.—To treat intelligently, it is absolutely essential that we should understand all the causes producing dental caries. Our aim should be to progress, giving improved methods a thorough test.

Dr. Brophy expressed himself as much pleased with the paper, several of the thoughts advanced being well worthy of close study. We need to know more about constitutional and local treatment. The old habit of extirpating a pulp, because nothing was known of intelligent treatment is altogether improper. To handle a case intelligently it is necessary first to ascertain constitutional susceptibilities, in order to determine whether treatment of the pulp will be successfnl or no. An injury to any part of the body, or slight abrasions of the skin, that suppurate for lack of a healthy tonicity are a pretty good indication that renders dubious any successful treatment of the pulp. It is his opinion that students of medicine should be better instructed on dental anatomy, pathology and therapy. These studies in connection with dental hygiene will give them a power that will save more teeth than all the dentists combined.

Dr. Talbert said that there are one or two points in the paper on the treatment of pulps by strong application of creosote and carbolic acid that he could not indorse. In his estimation, a mild course of treatment is more sensible, a one to five per cent. solution of carbolic acid being sufficient. To either creosote or carbolic acid oil of cloves is preferable. In the constitutional treatment of persons of plethoric habit, it is essential that the system be held in abeyance by the administration of cathartics, reducing the force and volume of the circulation. The blood then is sent to other regions better able to bear it. The temporary filling over an exposed pulp should remain for from six to twelve months, giving both tooth and pulp ample time to recuperate. Carbolic acid acts only as an antiseptic and not as a disinfectant, as we suppose, in applying to the roots of dead teeth.

Dr. Chappel.—Carbolic acid is used by the Illinois State Board of Health under the impression that it is a disinfectant.

Though it destroys animal organism, it can hardly be regarded as a disinfectant such as chlorine. Carbolic acid will not destroy purtrescence.

Dr. Brophy.—Carbolic acid will prevent the decomposition of animal matter, but when this matter is in a state of putrefaction, it will not remove or destroy the putrescent odor. Sulphuretted-hydrogen gas, when passed through liquid carbolic acid, will still retain its peculiar offensive odor.

Dr. J. Taft.—There are some offensive substances that will be destroyed by carbolic acid. A disinfectant acts by breaking up the putrescent combination. Su.phuretted hydrogen gas smells very bad indeed, but where the compound is destroyed by separating the sulphur from the hydrogen, neither one having a disagreeable odor, the smell of course disappears. In some instances carbolic acid is a good disinfectant. The value of chlorine as a disinfectant depends upon its affinity for hydrogen. This it extracts from theputrescent mass, and, in consequence, liberates oxygen in its nascent state. The oxygen then is really the disinfectant, the chlorine the means of producing it in its most active condition.

Dr. Chappel, in answer to a question about the preparation of Iodine, said that it should be prepared with especial reference to the use to which it is to be put. In its full strength it will act as an escharotic.

Dr. J. Taft.—In the treatment of pulps a mistaken idea as to its condition often leads to over treatment. In many simple cases, the only treatment required is the removal of the irritant. When a pulp has been exposed by awkwardness in excavating, there is no ache, nor inflammation, consequently neither stimuants, astringents, nor escharotics are required. This is, of course, true only of healthy persons, whose systems are in good condition. Simply cover the pulp to prevent the access and impingment of foreign bodies, that would create trouble by irritation. If a pulp has been exposed for some time, and there is more or less inflammation and pain, the treatment must be determined by a study of both local and constitutional symptoms. Both constitutional and accidental tendencies must enter into the account. Whatever is irritating must be thoroughly removed, and this applies to irritants of all kinds. They may be solid, fluid, or gaseous. The solids can be seen and removed with instruments, fluids and gases may be broken up by disinfectants and their power of irritating destroyed. Or, further changes may be prevented by use of antiseptics, thus obviating further irritation. After cleansing and disinfection, cover over the pulps and give nature a chance to restore the parts to their normal condition. Above all things, over treatment with escharotics is contra-indicated. New remedies and methods are continually being suggested and brought forward, and it is well to see what is in them. For a year past, instead of creosote, had been using the oil of eucalyptus and found it quite as good an antiseptic and a better disinfectant. In addition, its odor is more agreeable to the patient. It may be used in full strength and is decidedly more efficient than creosote in destroying the putrescent odor in roots. One application, full strength, thoroughly destroys the odor, when creosote will not.

Dr. Senaca Brown, upon being called, said he indorsed what had been said, but had nothing to add.

Dr. Brophy.—Dr. Taft, is not the oil of eucalyptus more acceptable to the patient than either creosote or carbolic acid?

Dr. J. Taft.—Yes, to the majority it is; though some few object to it.

Dr. F. Sage said his experience found it almost as objectionable as the other antiseptics.

Dr. Wm. Van Antwerp was requested to make some remarks pertaining to his experiments with the pawpaw. He replied that several years ago he determined to study closely the condition of exposed pulps, as they came under his observation. He found that in cases of long exposure, the inflammation induced proceeded to suppuration, part of the pulp (more or less) being in a sloughing condition. If that could be removed, and the remainder restored to a normal state, the result would be worth the trouble. He thought the slough could be removed by digesting it away. Pepsine was used with partial success.

One day, while away from home, he purchased a beefsteak, and in carrying it home the paper it was wrapped in became softened with the juice and tore away. Passing by a pawpaw thicket, he wrapped the meat in a number of the leaves. Several hours after reaching home, his wife called his attention to the peculiar appearance of the steak. He could not account for it; the meat looked queer and paler than when freshly cut, and having a sweet odor. He had recently introduced a gastric canula in a dog for experiment's sake, and recalled the appearance of meat taken from the dog's stomach, in a partially digested condition. It was identical, almost, with the appearance of his beefsteak, and it occurred to him that the steak was partially digested. It was cooked and found remarkably tender, proving to him that his surmise was correct. The idea of digesting away the slough in pulps being uppermost in his mind, he tried an impromptu extract of the rind of the pawpaw, and with partial success. He succeeded better with an aqueous extract of the leaves, they containing a larger proportion of the peptic matter. Had repeatedly tried to make an alcoholic extract of the leaves, but failed to preserve the peptic principle. The experiments with the pawpaw will be continued in the hope of ultimate success.

Dr. J. Taft.—As all practitioners differ in their methods of treating exposed pulps, he would suggest that all the members present give a synopsis of their method of treating such cases.

Dr. Sage said that he always allayed the irritation before capping the exposure. If the pulp is strangulated, he digests that part away. Has never had to remove a capping when the pulp was subjected to this preliminary treatment.

Dr. Cravens said that he did not know as much about exposed pulps now as he formerly thought he did. He does not believe that real, positive exposures are due to pathological conditions in more than 5 per cent. of all cases. He thinks the remaining 95 per cent. are the result of violence in some shape or other. The object for treatment of an exposure should be known before the treatment is begun; in other words, understand the case first. He thinks it is poor policy, in fact empirical, for an operator to do whatever A, B or C tells him, simply because they said so. Their methods applied to a specified case may have no bearing upon the requirements of the case at all. When part of a pulp is suppurating there is present that which endangers the life of the pulp, pus, and it is desirable to get rid of it. This pus, of course, is the result of an exalted inflammation. He thinks that once a pulp has been thoroughly congested it can not be saved alive, either by incision or by digesting away. The ultimate salvation of that pulp is very doubtful, indeed. He does not attempt to save a pulp until he has determined whether it has been violently congested or not. In the constant use of metallic and quill tooth-picks much violence is done to the teeth on the approximate surfaces. Especially is this true when either white or brown decay is present, many exposures being caused in this manner. If the pulp is in a fairly good condition, almost anything will do to cap with. (The doctor said mud from the street is good enough). In the great majority of cases success is due mainly to delicate manipulation. The capping must not impinge upon the pulp; as the pulp pulsates it is necessarily larger at one time than another, and it must have sufficient room to reach its fullest expansion. If not, both the patient and the dentist will soon hear from that tooth. Exudation may or may not take place from a congested pulp.

Dr. Clyde.—When a pulp is capped during congestion, and there is no exudation, is it not possible that the pulp will fill up the vacuum? He thought so; just as the mucous membrane does in the air-chamber of a plate.

Dr. Sage.—After an exposure the condition is not the same as at first, as the congestion and suppuration produce a greater or lesser amount of change. It has been his experience that but few pulps can be saved alive. In some cases the pulp dries up or mummifies, and without causing the least pain. It does not do this, however, unless something is done to stop suppuration. As a general thing, he finds it necessary to use pepsine before capping.

Dr. F. A. Hunter had, years ago, stopped capping pulps after the methods detailed to-day. He says that there are very few cases of absolute exposure when they are first seen, and nothing has been done to them. In most cases the pulp will be found congested and continually aching; if it is desirable to save them at all, devitalize the pulp with arsenic. In some cases it is well to leave a layer of softened dentine over the pulp, serving as a capping; if, however, the pulp is congested and aching, expose it and apply arsenic. It is next to impossible to cap a pulp so exactly that pressure will not be made upon it and yet leave no vacuum under the capping.

Dr. Clayton said that a very important point to observe in treating pulps, was not to remove the layer of odonto blasts immediately over the pulp. Osteo-dentine will not be formed if the natural covering of the pulp is taken away, or if the pulp has undergone suppuration. If the tooth is aching, and there is an acid reaction in the cavity, mop it out with bibulous paper; apply some alkaline substance and cover with cotton saturated in gum mastic. This can afterwards be removed and dry oxychloride or oxyphosphate can be substituted. He does not believe that secondary dentine will form over a pulp that is either suppurating or ulcerating.

SECOND DAY.—AFTERNOON SESSION.

This session was opened by Dr. Chappel, who detailed at some length many of the points in his paper, which appears elsewhere in the Register, and it is unnecessary to repeat them in the report. Dr. A. O. Rawls' paper on "The Possibilities of Success in the Treatment of Pulpless Teeth and Alveolar Abscess," was now in order. On motion the rules were suspended, so that the fifth subject could be taken up, giving Drs. Brophy and Talbot, of Chicago, an opportunity to illustrate on the black-board their methods of filling teeth. Dr. Betty then read his paper on "Filling Teeth with Cohesive Foil." The discussion that followed had no relevancy to the subject introduced, but was entertaining throughout.

Dr. Brophy began by stating that the subject of filling teeth is an old one, and that he had nothing new to offer. The material selected for filling should be adaptable and indestructible, so that it will resist the action of mastication. All that he had to say about gold, he said he took from Dr. Allport's method. The gold used by Dr. Allport is altogether non-cohesive, and though it wears away in some instances, it is thoroughly reliable. He proceeded to describe (illustrating by diagrams) the filling of a cavity on the proximal surface of a molar. Dr. Allport used to cut between the teeth to get sufficient space to work. Dr. Brophy prefers to obtain his space by spreading the teeth with cotton wedges. The lateral walls of the cavity are made straight up and down. A groove is cut at the cervical border and one in each lateral wall near the cervix. A large wad of gold, No. 4 non-cohesive, is first introduced and consolidated against the walls. The non-cohesive foil is carried up almost to the top of the cavity. A covering or capping of cohesive foil is put over the plug, in order to get a firm surface capable of resisting the action of mastication. The covering of cohesive foil is the only respect in which Allport's non-cohesive fillings, as now made, are better than those he made twenty-five years ago. The cylinders of non-cohesive foil are consolidated with the mallet in the same manner as cohesive foil. Dr. Allport also covered non-cohesive fillings on the buccal surface with cohesive foil, to prevent the plug wearing away by mastication. Cavities filled in the manner described above can be done in one fourth the time required to fill the same with cohesive foil.

Dr. French.---What is meant by the term "soft foil?"

Dr. Brophy.---The term "soft foil" is improperly used. *All* gold is soft; the difference is in the degree of cohesion as exhibited by the foil. Non-cohesive is the proper term to use in designating what is ordinarily called soft foil.

Dr. H. A. Smith.---The method of filling described by Dr. Brophy is a very practical one. One point to be observed is that the foil as it approaches the top, should gradually be made cohesive, so that the surface of cohesive foil will stay on. If the cohesive surface is put directly on the non-cohesive surface, it will break away, because the union is a mechanical one and not perfect.

Dr. Clayton said that in filling such cavities as Dr. Brophy described, he would use cylinders of soft foil made by rolling a ribbon on a broach and of sufficient length to cover the borders.

Dr. J. W. Jay, fully indorsed all that had been said about filling teeth with non-cohesive foil. He always uses this foil in proximal cavities wherever he can do it, covering with cohesive foil to get a resisting surface.

Dr. Talbot, endorsed the method as detailed by Dr. Clayton, especially as to making the border of the cavity round. He had always found great difficulty in doing this and designed a set of instruments for that especial purpose. The margins of the cavity should be rounded from the cervix to the crown.

Dr. Kempton, objected to the exaggeration of some of the gentlemen in speaking of the retaining pits used in cohesive foil filling. The pits are not intended to retain the filling as is erroneously supposed; they are for the purpose of holding firmly in place the first few pieces of gold. Neither, as has been stated, does it require two or three hours to fill with cohesive foil, a cavity having strong, thick walls, for in that case, the cavity could not be large.

Dr. Sage, noticed that Dr. Redman makes the lateral walls of proximal cavities square, instead of round as stated by Drs. Clayton and Talbot. The cylinders of soft foil are probably better when made flat.

THIRD DAY, MORNING---LAST SESSION.

The discussion of the sixth subject was resumed:

Dr. Clyde, said that the subject under discussion is Filling Teeth with Cohesive Foil and nothing was said upon that topic at all. He would like to say a word in favor of crystal gold. It is a good form of gold and does very well to begin a filling with, finishing off with foil. With its use undercuts are all that is necessary for retention, thus doing away with retaining pits. When working crystal gold, it is a mistake to use very small pointed instruments or to mallet too much. The pieces should be pressed into place, the instruments to have but shallow serrations and if not malleted too hard the gold will not pulverize. Teeth are very often injured by the enamel chipping off, where retaining pits are put, indicating too much force in condensing. We must not be too exclusive in our ideas about the form of gold to use for filling, but give each its due.

Dr. Clydes's remarks about crystal gold brought Dr. Leslie to his feet and the members were entertained for upwards of three quarters of an hour when he desisted upon Dr. Kempton's rising to a question of privilege. The subject was then passed and the society proceeded to elect officers, etc. Adjourned to meet first Wednesday in March, 1883.

